# Successful ventilation of two animals with a single ventilator: individualized shared ventilator setup in an in vivo model

**DOI:** 10.1186/s13054-020-03248-z

**Published:** 2020-08-27

**Authors:** Michiel Stiers, Tom Bleeser, Matthias Mergeay, Hannah Pinson, Luc Janssen, Tom Schepens

**Affiliations:** 1 Department of Emergency Medicine, St-Dimpna, J-B Stessensstraat 2, 2440 Geel, Belgium; 2 Department of Anesthesiology, UZ Leuven, Herestraat 49, 3000 Leuven, Belgium; 3 Department of Anesthesiology and Critical Care Medicine, St-Dimpna, J-B Stessensstraat 2, 2440 Geel, Belgium; 4 Applied Physics and Data Analytics, Vrije Universiteit Brussel, Pleinlaan 2, 1050 Brussels, Belgium; 5 Department of Critical Care Medicine, Antwerp University Hospital, University of Antwerp, Wilrijkstraat 10, 2650 Edegem, Belgium

Dear Editor,

As the ongoing COVID-19 crisis is spreading from developed into developing nations, a shortage of ventilators in ICUs can be expected during peak prevalence. Sharing a ventilator among patients has been put forward as a rescue solution [1, 2]; in this setting, the so-called pairing of patients with similar characteristics is needed [3–5]. We have developed a modified shared ventilator design that allows for individualization of tidal volumes and driving pressures, positive end-expiratory pressure (PEEP), and inspired oxygen fraction (FiO_2_) [6], which can thus substantially individualize the delivered breaths, removing the need of pairing (see Fig. 1).

**Fig. 1:**
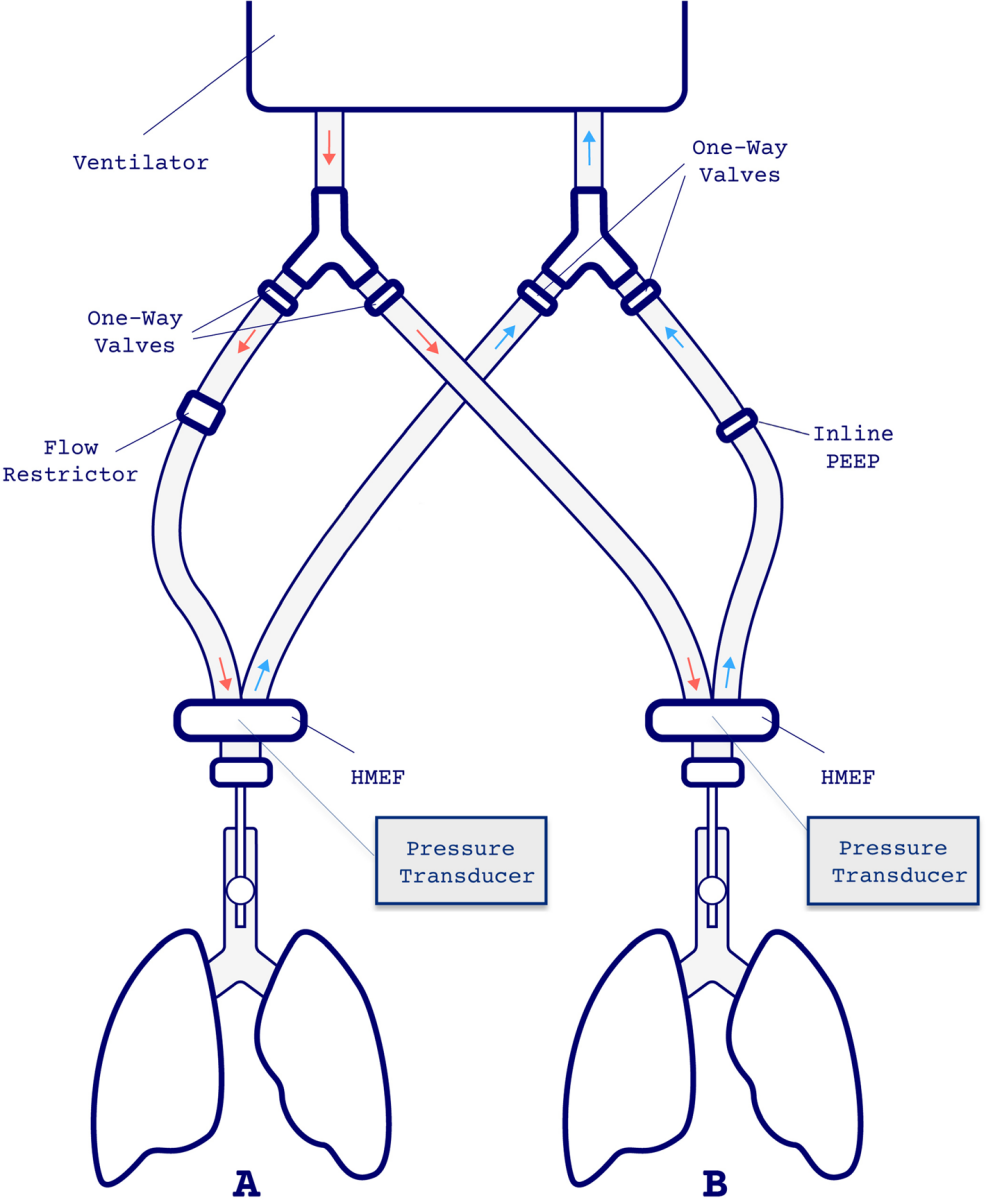
Individualized shared ventilator setup in an in vivo model. The ventilator we used was a Datex Ohmeda s/5 Aespire, T-connectors split expiratory and inspiratory circuits. The rotatory valve (Sisto-16RGA) is placed in the inspiratory limb to restrict pressure and thus tidal volume to the subject with the highest lung compliance, and an in-line PEEP valve (Intersurgical, ref. 2207000) is added to the expiratory limb of the circuit to set PEEP individually. Side-stream supply of additional oxygen can modulate the FiO_2_ that is delivered to each patient. One-way valves (Intersurgical, ref. 1921000) prevent cross-contamination

We have now successfully used this ventilator setup in an in vivo model in a pair of ventilated sheep with different lung compliance, further supporting the potential of this shared ventilator setup as a lifesaving intervention in a crisis setting.

After ethical approval, two healthy Swifter sheep (62 kg and 60 kg, 1 year old) received general anesthesia (buprenorphine-sevoflurane), intubation, arterial catheter, and a C-section. After baseline blood gas and respiratory mechanics measurements, both sheep were connected to a single ventilator. Animal 1 had a lung compliance of 38 ml cmH_2_O^− 1^, while animal 2 had a lung compliance of 28 ml cmH_2_O^− 1^, differences in compliance could be explained by their position. Ventilator settings and measurements are shown in Table 1. The targeted tidal volume of the shared ventilator was set by adding together the individual tidal volumes of animal 1 (600 ml) and 2 (800 ml), creating a combined tidal of 1400 ml. We measured individual airway pressures, with a fluid-air interfaced pressure transducer (Edwards Lifesciences, Irvine, USA), and individual end-tidal CO_2_ (etCO_2_) levels (see Fig. 1). We then partially closed the inspiratory flow for animal 1 until the measured etCO_2_ levels for each animal were similar to those measured at baseline. This titration was successfully achieved within a few breaths, and the total set tidal volume could be distributed accurately among the two animals. With the added in-line individual PEEP valve, animal 2 received a PEEP of 7 cmH_2_O, whereas the other received 3 cmH_2_O of PEEP. The individually measured airway pressures demonstrated that the set PEEP levels were successfully achieved for each animal. FiO_2_ could be adjusted as expected, with one animal receiving an FiO_2_ of ~ 0.3 and the other ~ 0.8 with added O_2_ to its breathing circuit during a short test period. Adequacy of ventilation and oxygenation in this setup was demonstrated with repeated blood gas measurements. Both PaCO_2_, PaO_2_, and pH values remained within normal range, thus we can assume that the individual tidal volumes before and after sharing the ventilator were similar. Hemodynamic parameters remained unchanged from baseline during the shared ventilator period. The animals were sacrificed after 3 h of mechanical ventilation.

**Table 1:** Ventilator settings and measurements of in vivo individualized shared ventilation

	Individual ventilation		Shared ventilator	
	Animal 1	Animal 2	Animal 1	Animal 2
Ventilator settings				
Tidal volume (ml)	600	800	1400	
PEEP (cmH_2_O)	3	4	3	7
FiO_2_	0.3	0.3	1.0	
I/E ratio	1:2	1:2	1:1.5	
Respiratory rate (min^−1^)	20	20	20	
Measured ventilatory values				
PEEP (cmH_2_O)	3	5	4	7
Ppeak vent (cmH_2_O)	18	32	31	
Ppeak circuit (cmH_2_O)			19	18
etCO_2_	31	33	32	29
Blood gas values				
pH	7.54	7.54	7.47	7.49
PaO_2_ (mmHg)	112	230	443	376
PaCO_2_ (mmHg)	31	30	39	36
Hemodynamic values				
BP (mmHg)	76/43	83/38	73/40	84/36

Table 1 shows the settings of the ventilator per animal and for the shared ventilator in a volume-controlled ventilation. In animal 2, inline PEEP was applied; in animal 1, the flow restriction with our valve was applied to distribute the pressures as desired among the two animals.

We demonstrated the potential to modulate delivered tidal volumes and pressures, PEEP and FiO_2_ in a shared ventilator setup in this in vivo model. The added ventilator circuit modifications are inexpensive and readily available or can be 3D-printed. This setup has allowed to safely ventilate a pair of animals with different lung compliance with a single ventilator, while monitoring and adjusting individual airway pressures and tidal volumes. However, I/E ratios and respiratory remain identical, and supplemental monitoring is required for safety reasons. We must stress that this setup is only to be used temporarily in a crisis setting while arranging for safer and more structural alternatives. The lung compliances were similar to what is frequently seen in ARDS. We think that this is a relevant step in the progressive development of a shared ventilator solution, but further research needs to be done to better understand its full potential in treating patients with COVID-19.

## Data Availability

All data generated or analyzed during this study are included in this published article and are shown in Table 1.

## Abbreviations

PEEP: Positive end-expiratory pressure
FiO_2_: Inspiratory fraction of oxygen
I/E: Inspiratory/expiratory time ratio
Ppeak: Peak pressure
vent: As measured by ventilator
Indiv: Individually measured on circuit
etCO_2_: End-tidal carbon dioxide
PaO_2_: Partial pressure of oxygen, arterial
PaCO_2_: Partial pressure of CO_2_, arterial
